# Replication Studies on Significant Differences in Personality Profiles of Securely and Insecurely Attached Psychotherapists and Dentists

**DOI:** 10.3389/fpsyg.2021.662828

**Published:** 2021-10-01

**Authors:** Burkhard Peter, Thomas G. Wolf

**Affiliations:** ^1^MEG-Stiftung München, Munich, Germany; ^2^Department of Restorative, Preventive and Pediatric Dentistry, School of Dental Medicine, University of Bern, Bern, Switzerland; ^3^Department of Periodontology and Operative Dentistry, University Medical Center of the Johannes Gutenberg-University Mainz, Mainz, Germany

**Keywords:** dentist, psychotherapist, homo hypnoticus, hypnosis, personality, attachment

## Abstract

This study contributes to the therapist variable in general and the personality profile of securely and insecurely attached psychotherapists and other healthcare professionals in particular. In a preceding study, it has been found that insecurely attached psychotherapists differ in nine personality styles from securely attached ones. The aim of the present study was to replicate these findings and to investigate whether they also apply to other health professions such as dentists. About 891 subjects from two German professional societies for hypnosis were surveyed online with a personality questionnaire [Personality Styles and Disorder Inventory (PSDI)] and an attachment questionnaire [Relationship Scale Questionnaire (RSQ)]. Since these subjects were interested in hypnosis and used it in their practice (HYP), 150 dentists without a hypnosis context (NONHYP) were studied as a control group with the same survey. The results of the preceding attachment study could be replicated: Insecurely attached healthcare professionals differed significantly from securely attached ones in the same nine (plus one, i.e., 10) personality styles if they use psychological methods including hypnosis. If they do not use psychological methods (like the NONHYP dentists), they differ in half of the personality styles. No within-sample and no between-sample differences have been found in the assertive/antisocial (AS) personality style. No within-sample differences have also been found in the conscientious/compulsive (ZW) and the intuitive/schizotypal (ST) personality styles. However, large between-sample differences were obvious in ZW and the ST. Both of the samples of the dentist were much more compulsive than the two psychotherapeutic samples. In addition, both of the HYP samples were much more schizotypal than the NONHYP samples. The latter is the general signature of those individuals who are interested in hypnosis and were metaphorically termed *homo hypnoticus*. It seems that AS, ZW, and ST are independent of attachment.

## Introduction

Since the early and pioneering work of Rogers (1957) and Frank (1961), the therapist variable came rather slowly into the focus of psychotherapy research. Part of the therapist variable is the personality of the psychotherapist, which in turn, is determined in part by his/her attachment style. Both variables, personality and attachment, contribute significantly to the ability of the therapist to form and maintain an effective psychotherapeutic relationship or working alliance, respectively, which is considered necessary for a good therapy process and outcome (Del Re et al., 2012; Flückiger et al., 2018). The newest research is about to gain more insight into the brain-to-brain concordance of this patient-clinician relationship (Ellingsen et al., 2020; Grahl et al., 2021). After a short review of the work of other researchers on the personality of the psychotherapist, Peter et al. (2017) presented a study on personality styles of 1,027 German-speaking psychotherapists. These therapists, surveyed with the Personality Styles and Disorders Inventory (PSDI, see Table 1; Kuhl and Kazén, 2009), showed especially low levels in contrast to the normative mean, of willful/paranoid (PN), spontaneous/borderline (BL), reserved/schizoid (SZ), and ambitious/narcissistic (NA) styles, with large effect sizes. Therefore, this sample of experienced clinicians (with an average of nearly 20 years of professional practice) was free from pathological personality styles and demonstrated a personality profile that the authors interpreted as being *necessary* for building a good therapeutic relationship. These psychotherapists were able to put their personal opinions aside, show empathy and appreciation, open themselves to the emotional experience of the patient, and provide a trusting relationship. Moderate differences, also below the normative mean, were found in the personality styles loyal/dependent (AB), critical/negativistic (NT), intuitive/schizotypal (ST), unselfish/self-sacrificing (SL), self-critical/avoidant (SU), passive/depressive (DE), and assertive/antisocial (AS). Peter et al. (2017) interpreted these styles as *helpful* for the professional social skills of psychotherapists, i.e., they were neither submissive nor critical, neither excessively helpful nor too self-critical, and neither passive nor too self-assertive.

**TABLE 1:** The 14 scales of the Personality Styles and Disorder Inventory (PSDI; Kuhl and Kazén, 2009).

PSDI-scale^*a*^	Example
PN willful/**paranoid**	“Most people mean well” (negatively coded)
BL spontaneous/**borderline**	“My feelings often change abruptly and impulsively”
SZ reserved/**schizoid**	“I always keep my distance to other people”
NA ambitious/**narcissistic**	“The idea of being a famous personality appeals to me”
AB loyal/**dependent**	“I need a lot of love and acceptance”
NT critical/negativistic	“I have frequently been persecuted by bad luck”
ST intuitive/**schizotypal**	“There are supernatural forces”
SL unselfish/self-sacrificing	“I am more concerned with other people’s worries than my own needs”
SU self-critical/**avoidant**	“Criticism hurts me quicker than it does to others”
DP passive/depressive	“I often feel low and feeble”
AS assertive/**antisocial**	“If people turn against me I can get them down”
HI charming/**histrionic**	“My good moods are very contagious to others”
RH optimistic/rhapsodic	“I am an invincible optimist”
ZW conscientious/**compulsive**	“Consistency and firm principles define my life”

*^*a*^DSM-5 or ICD-10 equivalents are in bold print.*

**FIGURE 1:**
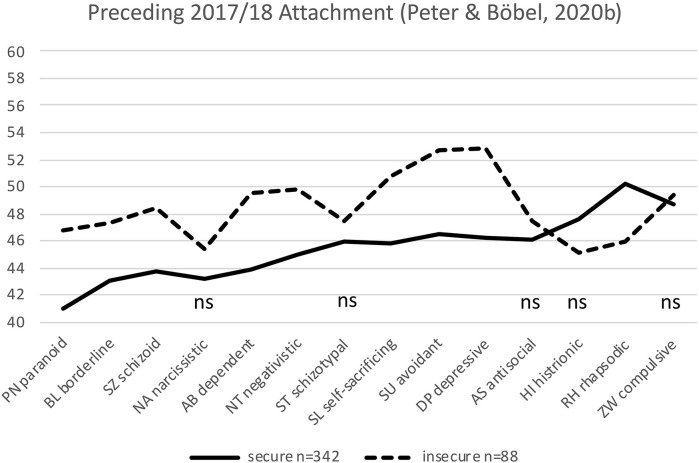
Significant differences in the preceding reference study by Peter and Böbel (2020b) between 342 securely and 88 insecurely attached psychological psychotherapists in nine of 14 personality styles. The non-significant differences are labeled ns (*t*-values are plotted on the *y*-axis; mean is *t* = 50; normal range is between *t* = 40 and *t* = 60; ns: not significant).

Peter and Böbel (2020b) investigated whether there may be differences between securely and insecurely attached psychotherapists. The concept of attachment originating from Bowlby (1969) states that the attachment behavior of a person is formed in early mother-child interaction and shapes the ability of a person to relate to others throughout life. Attachment styles are broadly categorized as secure or insecure. A person with a secure attachment style is thought to be confident and optimistic to others. An insecurely attached person—in contrast—is fearful, avoidant, or over-dependent toward others. It is, therefore, reasonable to expect that the therapeutic relationship may be affected by the attachment style of the therapist, and this may influence the therapeutic outcome. It has been proposed that securely attached psychotherapists may be more effective in psychotherapy than insecurely attached therapists, due to the impact of attachment style on the therapeutic alliance (Sauer et al., 2003; Black et al., 2005; Bruck et al., 2006; Mikulincer et al., 2013; Cologon et al., 2017; Heinonen and Nissen-Lie, 2019). Peter and Böbel (2020b) found that the 20% insecurely attached differed significantly from the 80% securely attached in nine of 14 personality styles in such a way that one could conclude that the insecurely attached were not good psychotherapists. In three of four styles in the first group of personality styles that Peter et al. (2017) had identified as *necessary* for a good psychotherapeutic relationship are PN, BL, and SZ; they showed significantly worse scores than the securely attached did. In addition, in the second group, which was considered *helpful* for a good psychotherapeutic relationship, the insecurely attached showed unfavorable scores in five out of seven styles. Similarly, in the third group, there was a significant difference in the optimistic/rhapsodic (RH) personality style. (For personality and attachment styles, see section “Survey Instruments” below, and Table 1.) These results of Peter and Böbel (2020b) (Figure 1) confirm those of Schauenburg et al. (2006), Sherry et al. (2007), and Both and Best (2017). However, the inference that the insecurely attached might be “bad” therapists was rejected because there were no therapy outcome data available with which to substantiate this assumption. Furthermore, these psychotherapists were consistently older and more professionally experienced colleagues (mean age of the two groups studied was 57.1; SD 9.39, and 52.2; SD 10.2), who may have compensated for their possible existing attachment-related disadvantages in the course of training and practical experience and through self-experience and supervision.

**FIGURE 2:**
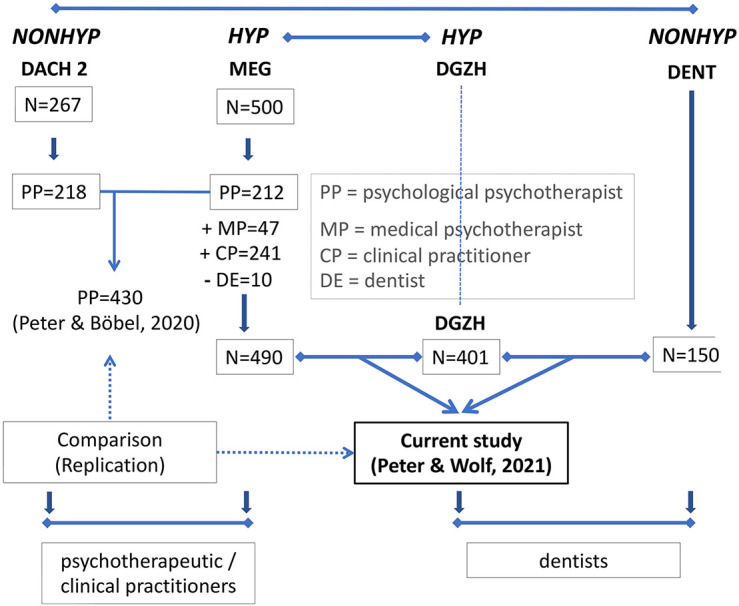
Flowchart illustrating the samples used for the current study.

Though significant, the data from Peter and Böbel (2020b) may have been incidental. Three further studies were conducted to replicate these results. One other focus of these further studies was to find confirmation data for a special personality profile of people who are interested in hypnosis and practice it in their healthcare professions. For people with such a personality profile, the term “hypnophilic” was first proposed by Peter (2018), followed by the metaphorical term *homo hypnoticus* (Peter and Böbel, 2020a). This issue, however, is relevant only insofar as two samples of the studies presented in this article were composed of healthcare professionals who were interested in and practiced hypnosis [the HYP samples Milton Erickson Society for Clinical Hypnosis (MEG) and German Society for Dental Hypnosis (DGZH)]. The other two samples were composed of professionals who were not interested and did not practice hypnosis (the NONHYP samples DACH 2 and DENT; see Figure 2; for details see below in the “Sample” section). Additionally, as the DACH 2 and the MEG samples in the majority were composed of psychotherapists and clinical practitioners, we were curious to find similar personality/attachment profiles in other healthcare providers with another profession, such as dentists. Therefore, we were mainly interested in whether the relationship between attachment and personality styles found in the preceding attachment study by Peter and Böbel (2020b) could be replicated in three other samples of the present research (HYP MEG, HYP DGZH, and NONHYP DENT). Accordingly, 14 personality scales were examined for the variable “attachment.” As an aside, to our knowledge, the present work is the first investigation to examine the personality and attachment styles of the dentist and the relationship between the two.

## Materials and Methods

### Sample

Two groups of healthcare professionals were contacted at the end of 2017 and again in February 2018 as a reminder. It could be assumed that all participants were interested in hypnosis: approximately 1,150 members of the German Society for Dental Hypnosis (DGZH) and approximately 3,500 participants of the listserv of the MEG in Germany. As too few DGZH members had responded in 2017/2018, the same call was repeated in early 2020. The participants of the MEG listserv were much more heterogeneous than the DGZH members in terms of their professions. The reason for this is that the MEG listserv is open to all who are generally interested in hypnosis/hypnotherapy, or the activities of the MEG in Germany, not just MEG members. DGZH and MEG were the two HYP samples. To get data from a NONHYP control group, about 1,100 dentists were also contacted by email in the second half of 2020 and early 2021, and asked to participate in an internet survey about the relationship between personality and attachment styles, using the online questionnaire software SoSci Survey (SoSci Survey GmbH, Munich, Germany). This is the NONHYP DENT sample. All three samples received the same questions *via* SoSci, except for specific questions about their respective occupations. As a thank you, they were presented with a link at the end of the questions that allowed them to download an article from the DACH psychotherapeutic practitioners for free (Peter et al., 2017).

HYP DGZH: A total of 418 DGZH members participated in the two runs of the survey, 246 in 2017/2018 and 217 in 2020. Those 45 who indicated in 2020 that they had already participated in 2017 were deleted from the dataset, so the 2020 dataset contained only 172 entries (172 + 246 = 418). There were 285 women (68.2%) and 133 men (31.8%) between 20 and 75 years of age (mean = 53.27; SD = 10.3). Three hundred eighty-eight were dentists, five were physicians, eight had other studies (mentioned here were stomatology, medicine and dentistry, oral surgery, maxillofacial surgery, gerontology, psychology, and chemistry), and only one person was without studies with training as a dental technician. Because of some missing figures of the Relationship Scale Questionnaire (RSQ) in 17 datasets, the final statistical calculation refers to only *N* = 401 (Figure 2).

HYP MEG: From the MEG listserv, 500 individuals responded (Figure 2). Because these MEG data were to be compared with those of DGZH dentists, the 10 dentists were deleted from the MEG file, leaving 490 records. There were 364 women (74.29%) and 126 men (25.71%) participants ranging in age from 22 to 82 years (mean = 52.22; SD = 10.2). Of these 490, 212 were psychological psychotherapists, and 47 were medical psychotherapists; the group also included 27 psychologists, 31 physicians, 56 psychotherapists according to the German Heilpraktikergesetz (law for healing practitioner), and 117 other professions. The last group, other professions, was very heterogeneous. It consisted, for example, of coaches, psychological counselors, pastors, students, psycho-oncologists, child and adolescent psychotherapists, family therapists or specialists in psychiatry and psychotherapy, and social pedagogues, most of whom were psychotherapists. The 56 psychotherapists according to the Heilpraktikergesetz were left in the database because 33 had completed a psychology degree and 17 another degree, and it could therefore be assumed that the license as Heilpraktiker was acquired only based on legal protection. Only six of these and nine from the 117 other professions had not completed any studies.

For reasons of care, one special feature of the MEG sample of this study should be pointed out. A subgroup from this MEG listserv dataset (*N* = 490), namely the 212 psychological psychotherapists, had already been used for the preceding attachment study by Peter and Böbel (2020b) (DACH 2 in Figure 2), which serves as a reference study for the present investigation. Thus, for the present study, we first determined whether these 212 psychological psychotherapists differed from the remaining 278 MEG participants of different professions. A *t*-test showed a significant difference [*t*(483, 74) = −3.51, *p* < 0.001] only for the personality style ST such that the psychological psychotherapists had lower ST scores than the rest of the participants did. Because there was no difference in all other styles, the data from the MEG sample of *N* = 490 were included as a whole in the present study.

NONHYP DENT: About 1,100 dentists were contacted from summer 2020 to spring 2021. Any indication of hypnosis was avoided in the invitation; 162 answered, 109 female (67.3%) and 53 male (32.87%) participants between 21 and 69 years of age (mean = 38; SD = 10.8). Their professional specialization was mixed (general dentistry, orthodontia, endodontics, oral surgery, and implantology). Because of some missing figures of the RSQ in 12 datasets, the final statistical calculation refers to only *N* = 150 (Figure 2).

### Survey Instruments

**FIGURE 3:**
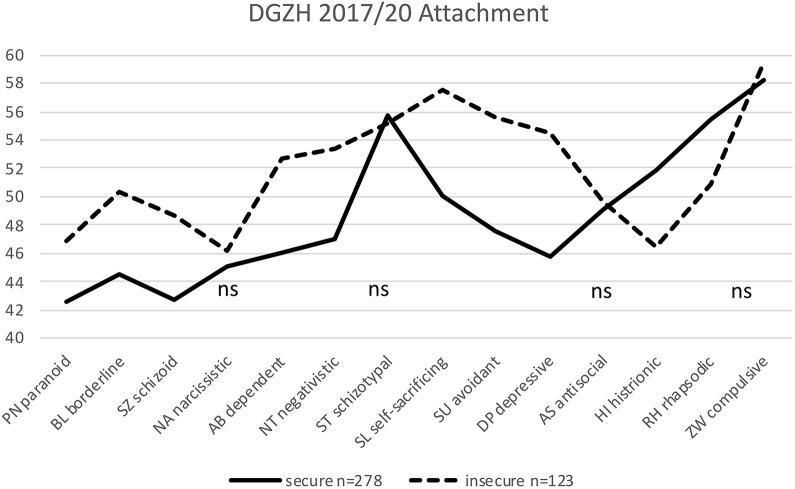
Significant differences between 278 securely and 123 insecurely attached HYP DGZH dentists in 10 of 14 personality styles. The non-significant differences are labeled ns (*t*-values are plotted on the *y*-axis; mean is *t* = 50; normal range is between *t* = 40 and *t* = 60; ns: not significant).

**FIGURE 4:**
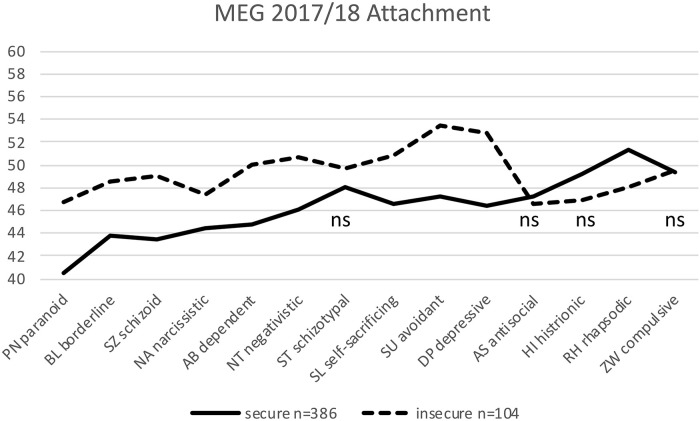
Significant differences between 386 securely and 104 insecurely attached HYP MEG participants in 10 of 14 personality styles. The non-significant differences are labeled ns (*t-*values are plotted on the *y*-axis; mean is *t* = 50; normal range is between *t* = 40 and *t* = 60; ns: not significant).

**FIGURE 5:**
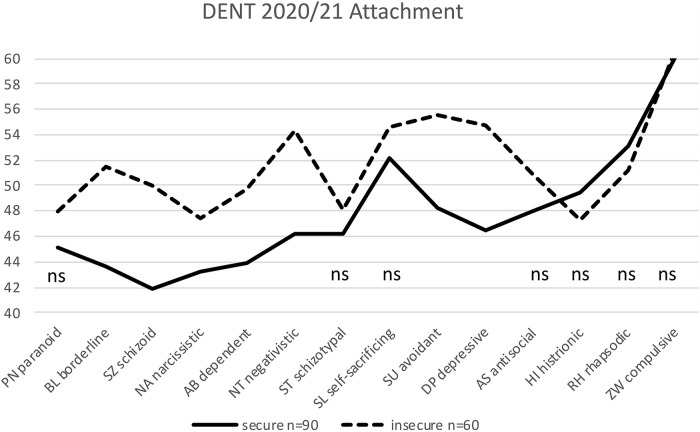
Significant differences between 90 securely and 60 insecurely attached dentists (NONHYP DENT) in seven of 14 personality styles. The non-significant differences are labeled ns (*t*-values are plotted on the *y*-axis; mean is *t* = 50; normal range is between *t* = 40 and *t* = 60; ns: not significant).

The PSDI by Kuhl and Kazén (2009) in its short form (PSDI-S) was used to assess personality styles. This inventory has been presented and described in detail two times in this journal (Peter et al., 2017; Peter and Böbel, 2020b). The PSDI is a self-reporting instrument that measures the relative expression of 14 personality styles. These are considered non-pathological equivalents of the personality disorders described in Diagnostic and Statistical Manual of Mental Disorders (DSM-IV) and International Statistical Classification of Diseases (ICD-10) (Table 1). In Figures 1, 3–5, the *t*-values of the PSDI are plotted on the *y*-axis. The mean *t*-value is 50, between 40 and 60 is the normal range. The German version of the Relationship Scales Questionnaire (RSQ) was used to assess attachment styles (Steffanowski et al., 2001; Steffanowski, 2001). The RSQ, a self-assessment scale of Griffin and Bartholomew (1994) based on the attachment theory of Bowlby (1969), has also been presented previously in this journal (Peter and Böbel, 2020b). In the present evaluation, the differential scoring and subsequent division of scores were reduced to only two factors, securely attached and insecurely attached, by combining the three scores for insecurely attached into one.

### Data Analysis

Data collected with SoSci Survey (SoSci Survey GmbH, Munich, Germany) were directly loaded into SPSS (IBM SPSS, version 27, IBM Corp., Armonk, NY, United States) and subsequently analyzed analogously according to Peter et al. (2017) and Peter and Böbel (2020b). The *t*-tests were used which are considered robust against violation of the normal distribution assumption. Because of testing all of the 14 scales of the PSDI, the significance level was set to *p* = 0.0036. Because of our interest in differences between secure and insecure participants, we computed within-sample comparisons only. Therefore, the few between-sample comparisons we examine in the discussion are only on a descriptive level.

### Ethics Statement

Participation was voluntary and had neither advantages nor disadvantages for the study participants. By answering the questionnaire, participants gave their written consent to the processing of their irreversibly anonymized data; they received no compensation. No formal approval for this type of study by the local ethics committee was required because the data of the study participants were collected and processed under irreversibly anonymized conditions. This is in accordance with the Swiss Human Research Act [810.30 Federal Law on Research Involving Human Subjects, Human Research Act (HRA)]. All procedures were performed in accordance with the 1964 Declaration of Helsinki and its subsequent amendments and the ethical standards of the local research commission. In addition, all study participants were of legal age and gave written informed consent for the processing of their data for research purposes.

## Results

HYP DGZH: The 278 securely attached DGZH dentists differed significantly from the 123 (31%) insecurely attached DGZH dentists in 10 out of 14 personality styles, but not in the NA, ST, AS, and conscientious/compulsive (ZW) styles (Table 2 and Figure 3).

**TABLE 2:** Test statistics for differences between 278 securely and 123 insecurely attached HYP DGZH dentists in 14 personality styles of the PSDI.

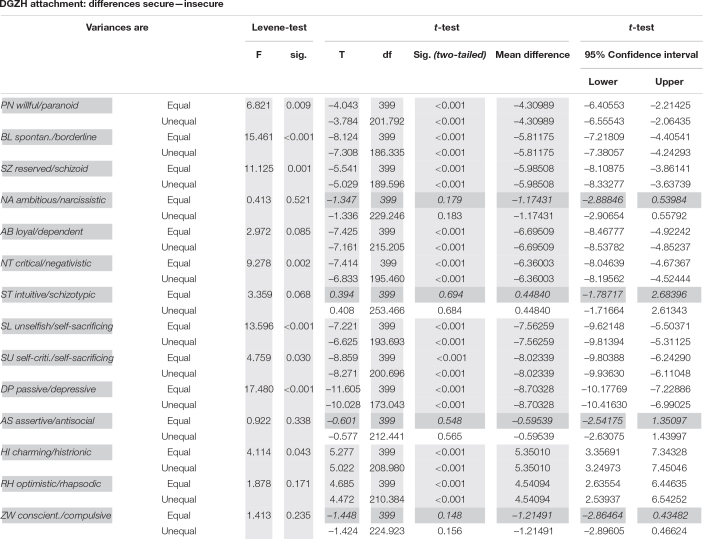

*p(0.5/14) = 0.0036; non-significant results (NA, ST, AS, and ZW) gray and italic.*

HYP MEG: The 386 securely attached MEG participants differed significantly from the 104 (21%) insecurely attached MEG participants in 10 out of 14 personality styles, but not in the ST, AS, charming/histrionic (HI), and ZW styles (Table 3 and Figure 4).

**TABLE 3:** Test statistics for the differences between 386 securely and 104 insecurely attached HYP MEG participants in 14 personality styles of the PSDI.

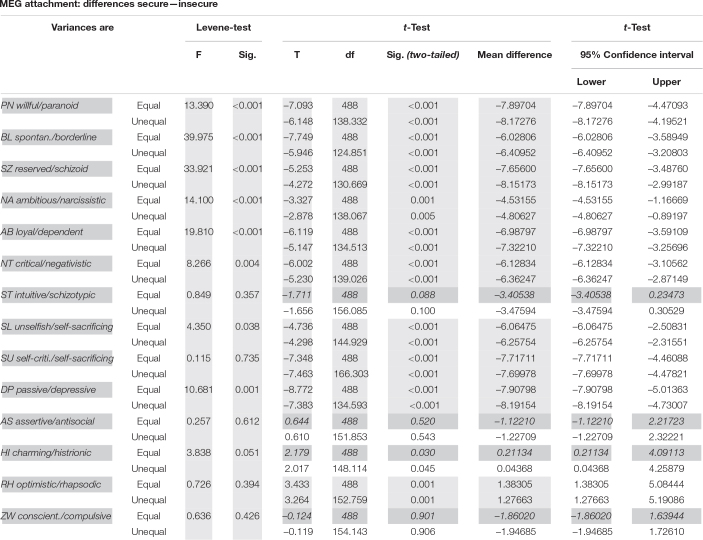

*p(0.5/14) = 0.0036; non-significant results (ST, AS, Hi and ZW) gray and italic.*

NONHYP DENT: The 90 securely attached dentists differed significantly from the 60 (40%) insecurely attached dentists in seven out of 14 personality styles, but not in the PN, ST, SL, AS, HI, RH, and ZW styles (Table 4 and Figure 5).

**TABLE 4:** Test statistics for the differences between 90 securely and 60 insecurely attached NONHYP DENT participants in 14 personality styles of the PSDI.

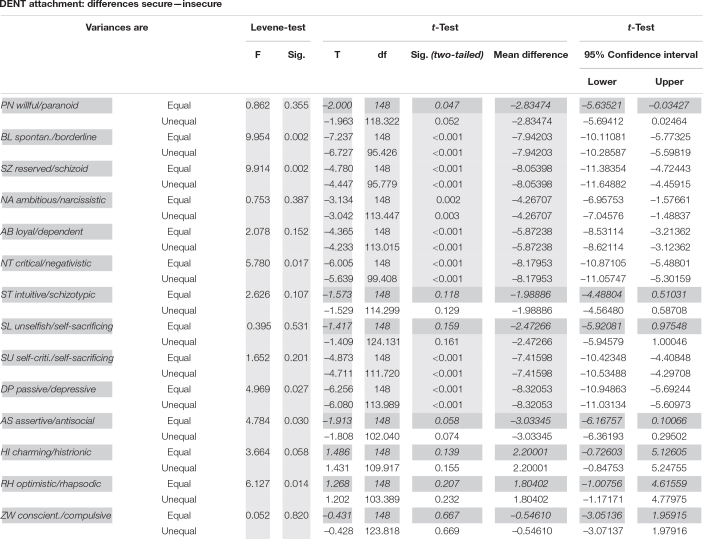

*p(0.5/14) = 0.0036; non-significant results (PN, ST, SL, AS, Hi, RH, and ZW) gray and italic.*

## Discussion

Attachment is an essential aspect of the therapeutic relationship on the part of both patients (Levy et al., 2018) and therapists (Steel et al., 2018), which has only recently begun to be scientifically researched (Slade and Holmes, 2018). The sometimes-contradicting research results can be explained, by, among other things, the fact that two multiplied by two attachment styles (secure and insecure, patient and therapist) encounter each other in the therapeutic interaction, which then behaves symmetrically or complementarily to each other in different ways and are more or less well matched to the respective therapeutic process. For example, in a review study, Degnan et al. (2016) demonstrated that securely attached therapists formed a stronger therapeutic alliance with their patients and thus achieved better therapeutic outcomes. Steel et al. (2018) showed that relationship quality, negative countertransference, empathy skills, and coping with problems strongly depend on the attachment style of therapists. With the present study, we aim to contribute to this still under-researched topic.

The purpose of the present study was to investigate the relationship between personality and attachment styles in three samples of professionals working in the healthcare system to find personality and attachment profiles similar to the preceding attachment study of Peter and Böbel (2020b) who then surveyed only psychotherapists. The MEG sample of the present study comprised also psychotherapists, and other clinical practitioners. The other two samples of the present study comprised only dentists. Aside from this distinction between psychotherapeutic/clinical practitioners and dentists, there was another distinction in the sample composition: Two of the three samples in the current study were members or participants of two German hypnosis societies—dentists of the German Society of Dental Hypnosis (DGZH) and psychotherapeutic practitioners of the Milton Erickson Society for Clinical Hypnosis Germany (MEG). These were the two HYP groups. The third sample consisted of dentists who were contacted as a NONHYP control group, i.e., without any association to hypnosis. This NONHYP criterion pertained also to the preceding attachment study of Peter and Böbel (2020b) (see Figure 2). This differentiation between HYP and NONHYP participants refers to a different research question that is covered in detail in another paper. It is not relevant for the present study with one exception, which will be discussed below.

As mentioned in the description of the samples above, half of the total MEG sample used here (212 out of 490) were included in the preceding attachment study of Peter and Böbel (2020b), which served as the reference study. It is, therefore, not surprising that the percentage of 21% insecurely attached in the current MEG sample is close to the 20% in the preceding of Peter and Böbel (2020b) attachment study, both of which are similar to the percentages in a German (Taubner et al., 2014) and a North American (Fleischman and Shorey, 2016) studies. Among DGZH dentists, the percentage of insecurely attached is 10% higher, at 31%, and that of the dentists of the NONHYP DENT sample is another 10% higher, at 40%.

According to our hypothesis, we aimed to test whether similar within-sample differences between the securely and insecurely attached can also be found in other groups of therapeutic professions, as in the preceding attachment study of Peter and Böbel (2020b). This hypothesis was well confirmed. In view of Figures 3–5, it is apparent that the results of the preceding attachment study of Peter and Böbel (2020b) (Figure 1) could be replicated. All four samples, by and large, show similar profiles (Figures 1, 3–5) with the four *necessary* and seven *helpful* personality profiles (according to Peter et al., 2017) of the insecurely attached being “worse,” i.e., above that of the securely attached, and being below, i.e., also “worse” in the HI and RH personality styles. With almost the same few exceptions, the first two samples of the current study (MEG and DGZH) show the same significant differences in personality styles between the securely and insecurely attached as in Peter and Böbel (2020b). As noted, this is not surprising for the MEG sample; however, for the DGZH sample, it is quite a remarkable result. Formally, it points to the high reliability and constructs validity of the applied tests PSDI and RSQ. Regarding content it suggests that professionals in the healthcare system who work with patients by using special psychological methods (psychotherapy and/or hypnosis) for modifying affect, thinking and behavior show similar personality profiles that additionally differ whether these professionals owe a secure or unsecured attachment style.

No differences—similar to Peter and Böbel (2020b)—show up in the DGZH sample in the style NA; thus, the DGZH dentists are all equally NA, completely independent of their attachment style. In this, they differ from the MEG sample, in which the securely attached are significantly less ambitious than the insecurely attached are. In the MEG sample, there is no difference in the HI style. The main exceptions, however, in which no differences were shown between secure and insecure participants in all samples concern the styles ST, ZW, and AS. In these styles (labeled X in Table 5), the three samples of the present study and that of Peter and Böbel (2020b) show no differences between the securely and insecurely attached. In our view, it is obvious that these three personality styles are independent of attachment. Even not being a within-group difference, the personality style ST shows a prominent difference between the dentists whether they are interested in and use hypnosis (the HYP DGZH sample) or not (the NONHYP DENT sample). This is the significant feature of the *homo hypnoticus*: The difference of around eight *t*-values between the HYP DGZH (Figure 3) and NONHYP DENT (Figure 5) participants was found to be highly significant in our data (not included in this study) and parallels equal findings in other samples (Peter and Böbel, 2020a). In the personality style that is probably most important for dentists in the practice of their profession—i.e., ZW—all securely and insecurely attached dentists do not differ at all within groups (DGZH and DENT), with very high values overall. Yet, they differ by about 10 *t*-values from the psychotherapeutic sample (DACH 2 and MEG) whose participants show only medium values overall in ZW style in the securely a well as insecurely attached. Finally, no differences are seen in the style AS, neither between the securely and insecurely attached nor between the four different samples; all values are just below the average norm value of 50. This is reassuring but self-evident; all healthcare professionals should not be too assertive or even aggressive regardless of whether they are securely attached or not.

**TABLE 5:** In the personality styles labeled **X**, there is no difference between the securely and insecurely attached participants of the original study of Peter and Böbel (2020b) (Figure 1) and of the three samples of the present study.

	Peter and Böbel (2020b)	Present study			
		DGZH	MEG	DENTists
Willful/**paranoid (PN)**				**X**
Spontaneous/**borderline (BL)**				
Reserved/**schizoid (SZ)**				
Ambitious/**narcissistic (NA)**	**X**	**X**		
Loyal/**dependent (AB)**				
Critical/negativistic (NT)				
Intuitive/**schizotypic (ST)**	**X**	**X**	**X**	**X**
Unselfish/self-sacrificing (SL)				**X**
Self-critical/**self-sacrificing (SU)**				
Passive/depressive (DP)				
Assertive/**antisocial (AS)**	**X**	**X**	**X**	**X**
Charming/**histrionic (HI)**	**X**		**X**	**X**
Optimistic/rhapsodic (RH)				**X**
Conscientious/**compulsive (ZW)**	**X**	**X**	**X**	**X**

*In all other personality styles, the differences between the securely and insecurely attached are significant (see Figures 3–5).*

Looking more specifically from a descriptive level at the profiles of the two dentist samples DGZH and DENT (Figures 3, 5) (with the exception of the just mentioned ST style), these profiles are more similar to each other as, in comparison, the two psychotherapeutic samples MEG and the preceding DACH 2 of Peter and Böbel (2020b) (Figures 1, 4) that again are also similar to each other. The differences in samples of the dentist between the securely and insecurely attached participants are larger than that of samples of the psychotherapist.

Peter and Böbel (2020b) conjectured that insecure attachment may be a hindrance for professionals in the healthcare system to establish a good therapeutic relationship. However, unlike with psychotherapists and hypnotherapists, for whom the therapeutic relationship is an essential part of their professional work (Peter and Revenstorf, 2017; Flückiger et al., 2018; Heinonen and Nissen-Lie, 2019), this need not be a disadvantage for the *lege artis* practice of dentistry in general, but it could be a disadvantage for dentists when performing hypnosis in their dental practice. Analogous to Peter and Böbel, 2020b) and because we also had no concrete data of therapy results in this study with which the accuracy of this assumption could have been proven, we can only refer to the average age of our participants (DGZH: 53.27 years; MEG: 52.22 years), which indicates many years of professional practice through which personality and attachment style-related disadvantages can be compensated through psychotherapy, self-experience, and supervision. This concerns not only psychotherapeutic/clinical professionals but also dentists if they use hypnosis. Even if attachment styles may be stable for a lifetime—a statement which is increasingly questioned (Girme et al., 2018; Fraley, 2019; Luyten et al., 2021)—at least coping with an unsecured attachment may be learned by psychotherapy, education, and training (Taylor et al., 2015; Buchheim et al., 2017; Rizou and Giannouli, 2020). Whether this is possible in the process of psychotherapy education must be proved by further studies. Applying PSDI and RSQ or other adequate measurements at the beginning and the end of psychotherapy education curricula could help to answer this question.

## Limitations

First, we must state that the choices of samples, psychotherapists/clinical practitioners and dentists, do correspond to the respective professions of the two authors. This may be considered a kind of selection bias (Peter and Roberts, in press) that, however, is not significant for the result and its interpretation of the present study. Second, the use of self-rating scales in psychological research is generally problematic. This caveat should be applied also to the present study. For example, we do not know whether the differences in the percentages of the insecurely attached participants between the HYP psychotherapeutic/clinical practitioners (MEG: 21%), the HYP dentists (DGZH: 31%), and the NONHYP dentists (DENT: 40%) are valid personality differences or whether they are caused by a kind of self-deceiving bias insofar as the dentists are rather naïve toward the special meanings of the particular items of the RSQ in contrast to the psychotherapeutic practitioners who, if only intuitively, know which answer would better fit a special professional image. On a different note, one can consider the differences in the ages. With a mean age of 38, the DENT sample is about 14 years younger than the participants of all other samples. If the assumption made above is reasonable that practice of psychological methods, self-experience, and supervision can compensate or probably change disadvantaged personality and attachment styles, then the professionals of the three psychotherapeutic/clinical samples achieved some success in this regard. In contrast to Peter and Böbel (2020a), we did not perform a gender breakdown because the preceding study of Peter and Böbel (2020b) to which we made the comparison did not do so. Additionally, because of the low percentage of male participants, we would have had too few numbers per cell for the insecurely attached men. Finally, as the same differences between securely and insecurely attached, and another three differences have been found in the control group of the 150 NONHYP dentists, it would be worthwhile to find out by other studies whether this now established personality-attachment profile is specific just for healthcare providers or is a general pattern, which can be found in other occupational groups, too.

## Conclusion

Regarding the main theme of the present study, i.e., attachment, it can be concluded that the results of the preceding attachment study of Peter and Böbel (2020b) have been replicated: Insecurely attached healthcare professionals differed significantly from securely attached ones in 10 out of 14 personality styles if they use psychological methods including hypnosis. If they do not use psychological methods (like the NONHYP DENT), they differ in less, i.e., half of the personality styles. Among the exceptions where no within-group differences have been found are the AS and the ZW personality styles. This seems reasonable to us as explained above. However, among these exceptions of no within-group differences are also the primary signature of the *homo hypnoticus* (Peter and Böbel, 2020a, #69710), the ST personality style. This seems intriguing to us and calls for a more in-depth interpretation, which would go beyond the scope of this article. At least, there are strong indications that these three personality styles, AS, ST, and ZW, are independent of the attachment type of healthcare providers.

## Data Availability Statement

The data analyzed in this study is subject to the following licenses/restrictions: General European Data Protection Regulation (GDPR) of May 25th 2018. Requests to access these datasets should be directed to BP, Burkhard-Peter@t-online.de.

## Ethics Statement

Ethical review and approval was not required for the study on human participants in accordance with the local legislation and institutional requirements. The patients/participants provided their written informed consent to participate in this study.

## Author Contributions

BP and TW designed and planned the study. Both were in charge of the experimental and BP of the statistical conduction of the investigation. BP prepared the first draft of the manuscript. TW revised the first draft of the manuscript. Both authors read and approved the final manuscript.

## Conflict of Interest

The authors declare that the research was conducted in the absence of any commercial or financial relationships that could be construed as a potential conflict of interest.

## Publisher’s Note

All claims expressed in this article are solely those of the authors and do not necessarily represent those of their affiliated organizations, or those of the publisher, the editors and the reviewers. Any product that may be evaluated in this article, or claim that may be made by its manufacturer, is not guaranteed or endorsed by the publisher.

## References

[B1] Black S. Hardy G. Turpin G. Parry G. (2005). Self-reported attachment styles and therapeutic orientation of therapists and their relationship with reported general alliance quality and problems in therapy. *Psychol. Psychother.* 78 363–377. 10.1348/147608305X43784 16259852

[B2] Both L. E. Best L. A. (2017). A comparison of two attachment measures in relation to personality factors and facets. *Pers. Individ. Diff.* 112 1–5.

[B3] Bowlby J. (1969). *Attachment and Loss: Vol. 1 Attachment.* New York, NY: Basic Books.

[B4] Bruck E. Winston A. Aderholt S. Muran J. C. (2006). Predictive validity of patient and therapist attachment and introject styles. *Am. J. Psychother.* 60 393–406. 17340948 10.1176/appi.psychotherapy.2006.60.4.393

[B5] Buchheim A. Hörz-Sagstetter S. Doering S. Rentrop M. Schuster P. Buchheim P. (2017). Change of unresolved attachment in borderline personality disorder: RCT study of transference-focused psychotherapy. *Psychother. Psychos.* 86 314–316. 10.1159/000460257 28903103

[B6] Cologon J. Schweitzer R. D. King R. Nolte T. (2017). Therapist reflective functioning, therapist attachment style and therapist effectiveness. *Adm. Policy. Ment. Health* 44 614–625. 10.1007/s10488-017-0790-5 28132188

[B7] Degnan A. Seymour-Hyde A. Harris A. Berry K. (2016). The role of therapist attachment in alliance and outcome: a systematic literature review. *Clin. Psychol. Psychother.* 23 47–65. 10.1002/cpp.1937 25445258

[B8] Del Re A. C. Flückiger C. Horvath A. O. Symonds D. Wampold B. E. (2012). Therapist effects in the therapeutic alliance–outcome relationship: a restricted-maximum likelihood meta-analysis. *Clin. Psychol. Rev.* 32 642–649. 10.1016/j.cpr.2012.07.002 22922705

[B9] Ellingsen D. M. Isenburg K. Jung C. Lee J. Gerber J. Mawla I. (2020). Dynamic brain-to-brain concordance and behavioral mirroring as a mechanism of the patient-clinician interaction. *Sci. Adv.* 6:eabc1304. 33087365 10.1126/sciadv.abc1304 PMC7577722

[B10] Fleischman S. Shorey H. S. (2016). The relationships between adult attachment, theoretical orientation, and therapist-reported alliance quality among licensed psychologists. *Psychother. Res. J. Soc. Psychother. Res.* 26 95–105. 10.1080/10503307.2014.947390 25118564

[B11] Flückiger C. Del Re A. C. Wampold B. E. Horvath A. O. (2018). The alliance in adult psychotherapy: a meta-analytic synthesis. *Psychotherapy* 55 316–340. 10.1037/pst0000172 29792475

[B12] Fraley R. C. (2019). Attachment in adulthood: recent developments, emerging debates, and future directions. *Annu. Rev. Psychol.* 70 401–422. 10.1146/annurev-psych-010418-102813 30609910

[B13] Frank J. D. (1961). *Persuasion and Healing. A Comparative Study of Psychotherapy.* Baltimore: Johns Hopkins University Press.

[B14] Girme Y. U. Agnew C. R. VanderDrift L. E. Harvey S. M. Rholes W. S. Simpson J. A. (2018). The ebbs and flows of attachment: within-person variation in attachment undermine secure individuals‘ relationship wellbeing across time. *J. Pers. Soc. Psychol.* 114 397–421. 10.1037/pspi0000115 29189026 PMC5820166

[B15] Grahl A. Anzolin A. Isenburg K. Lee J. Ellingsen D.-M. Jung C. (2021). Brain and behavioral correlates of the patient-clinician relationship: a longitudinal fMRI hyper-scanning study of chronic pain patients. *J. Pain* 22:602. 10.1016/j.jpain.2021.03.097

[B16] Griffin D. Bartholomew K. (1994). Models of the self and other - Fundamental dimensions underlying measures of adult attachment. *J. Pers. Soc. Psychol.* 67 430–435.

[B17] Heinonen E. Nissen-Lie H. A. (2019). The professional and personal characteristics of effective psychotherapists: a systematic review. *Psychother. Res.* 30 417–432. 10.1080/10503307.2019.1620366 31122157

[B18] Kuhl J. Kazén M. (2009). *Persönlichkeits-Stil- und Störungs-Inventar (PSSI). Manual [Personality Style and Disorder Inventory (PSDI).* Göttingen: Hogrefe.

[B19] Levy K. N. Kivity Y. Johnson B. N. Gooch C. V. (2018). Adult attachment as a predictor and moderator of psychotherapy outcome: a meta-analysis. *J. Clin. Psychol.* 74 1996–2013. 10.1002/jclp.22685 30238450

[B20] Luyten P. Campbell C. Fonagy P. (2021). Rethinking the relationship between attachment and personality disorder. *Curr. Opin. Psychol.* 37 109–113. 33385979 10.1016/j.copsyc.2020.11.003

[B21] Mikulincer M. Shaver P. R. Berant E. (2013). An attachment perspective on therapeutic processes and outcomes. *J. Pers.* 81 605–616. 10.1111/j.1467-6494.2012.00806.x 22812642

[B22] Peter B. (2018). 40 Jahre M.E.G. Kommt jetzt die Midlifecrisis? Zum Schizotypie- und Laien-Problem der Hypnose [40 years M.E.G. Is midlife crisis now appoaching? On the schizotypy- and lay-problem of hypnosis]. *Hypnose ZHH* 13 5–28.

[B23] Peter B. Böbel E. (2020b). Significant differences in personality styles of securely and insecurely attached psychotherapists. Data, reflections and implications. *Front. Psychol.* 11:611. 10.3389/fpsyg.2020.00611 32373012 PMC7186447

[B24] Peter B. Böbel E. (2020a). Does the Homo hypnoticus exist? Personality styles of people interested in hypnosis. *Int. J. Clin. Exp. Hypnosis* 68 348–370. 10.1080/00207144.2020.1756294 32436769

[B25] Peter B. Böbel E. Hagl M. Richter M. Kazén M. (2017). Personality styles of German-speaking psychotherapists differ from a norm, and male psychotherapists differ from their female colleagues. *Front. Psychol.* 8:840. 10.3389/fpsyg.2017.00840 28596747 PMC5443143

[B26] Peter B. Revenstorf D. (2017). Rapport und therapeutische Beziehung in der Hypnotherapie [Rapport and therapeutic alliance in hypnotherapy]. *Verhaltenstherapie Verhaltensmedizin* 38 424–447.

[B27] Peter B. Roberts L. (in press). Hypnotizability norms may not be representative of the general population: potential sample and self-selection bias considerations. *Int. J. Clin. Exp. Hypn*. 10.1080/00207144.2021.2003694 35020571

[B28] Rizou E. Giannouli V. (2020). An exploration of the experience of trainee integrative psychotherapists on therapeutic alliance in the light of their attachment style. *Health Psychol. Res.* 8:9177. 10.4081/hpr.2020.9177 33553790 PMC7859965

[B29] Rogers C. (1957). The necessary and sufficient conditions of therapeutic personality change. *J. Consult. Psychol.* 22 95–103. 10.1037/h0045357 13416422

[B30] Sauer E. M. Lopez F. G. Gormley B. (2003). Respective contributions of therapist and client adult attachment orientations to the development of the early working alliance: a preliminary growth modeling study. *Psychother. Res.* 13 371–382. 21827249 10.1093/ptr/kpg027

[B31] Schauenburg H. Dinger U. Buchheim A. (2006). Bindungsmuster von Psychotherapeuten. [Attachment patterns in psychotherapists]. *Z. Psychosom. Med. Psychother.* 52 358–372. 17156605 10.13109/zptm.2006.52.4.358

[B32] Sherry A. Lyddon W. J. Henson R. K. (2007). Adult attachment and developmental personality styles: an empirical study. *J. Counsel. Dev.* 85 337–348.

[B33] Slade A. Holmes J. (2018). Attachment and psychotherapy. *Curr. Opin. Psychol.* 25 152–156. 10.1016/j.copsyc.2018.06.008 30099208

[B34] Steel C. Macdonald J. Schroder T. (2018). A systematic review of the effect of therapistś internalized models of relationships on the quality of the therapeutic relationship. *J. Clin. Psychol.* 74 5–42. 10.1002/jclp.22484 28505384

[B35] Steffanowski A. (2001). *Konstruktion und Validierung Von Bindungsdiagnostischen Skalen [Construction and Validation of Attachment Scales].* Heidelberg: SAS Universität.

[B36] Steffanowski A. Oppl M. Meyerberg J. Schmidt W. W. Wittmann W. W. Nübling R. (2001). “Psychometrische überprüfung einer deutschsprachigen version des relationship scales questionaire (RSQ),” in *Störungsspezifische Therapieansätze - Konzepte und Ergebnisse*, ed. Bassler M. (Gießen: Psychosozial).

[B37] Taubner S. Ulrich-Manns S. Klasen J. Curth C. Möller H. Wolter S. (2014). Innere Arbeitsmodelle von Bindung und aversive Kindheitserfahrungen bei Psychotherapeuten in Ausbildung. [Inner working models of attachment and aversive childhood experiences of psychotherapists in training]. *Psychother. Forum* 19 2–12.

[B38] Taylor P. Rietzschel J. Danquah A. Berry K. (2015). Changes in attachment representations during psychological therapy. *Psychother. Res.* 25 222–238. 10.1080/10503307.2014.886791 24559454

